# Hyperthermophilic pretreatment composting can reduce ammonia emissions by controlling proteolytic bacterial community and the physicochemical properties

**DOI:** 10.1186/s40643-023-00659-y

**Published:** 2023-07-08

**Authors:** Ying Huang, Yuehong Chen, Hongying Huang, Ghulam Mustafa Shah, Jiujun Lin, Meiling Yan, Chengbao Guo, Xu Xiao

**Affiliations:** 1 Nanjing Institute of Agricultural Sciences in Jiangsu Hilly Area, No. 6 Xianyin South Road, Qixia District, Nanjing, 210046 Jiangsu Province China; 2 Institute of Agricultural Resources and Environment Jiangsu Academy of Agricultural Sciences, Jiangsu Academy of Agricultural Sciences, No. 50 Zhongling Street, Xuanwu District, Nanjing, 210014 Jiangsu Province China; 3 Jiangsu Collaborative Innovation Center for Solid Organic Waste Resource Utilization, Nanjing, 210014 Jiangsu Province China; 4 Department of Environmental Sciences, COMSATS University Islamabad Vehari Campus, Vehari, 61100 Pakistan

**Keywords:** Hyperthermophilic pretreatment composting, NH_3_ mitigation, The *sub* and *npr* proteolytic bacterial community, Protease activity

## Abstract

Proteolysis is the rate-limiting step in the mineralization of organic nitrogen into ammonium (NH_4_^+^) and thereby the ammonia (NH_3_) released during the composting. However, the dynamics of bacterial proteolytic communities related to NH_3_ emissions during the composting systems are mostly unknown. This study aimed to examine and compare the effects of hyperthermophilic pretreatment composting (HPC) and traditional composting (TC) methods on (i) the difference of NH_3_ loss and nitrogenous compounds; (ii) the dynamics of the proteolytic bacterial community involved in the proteolysis and (iii) the correlation between the proteolytic bacterial community, biophysiochemical characteristics and NH_3_ loss. Results revealed that the HPC decreased NH_3_ loss by 42% as compared to TC during 60-day composting period. This was accompanied with an inhibitory effect on protease activity in the HPC where the relative abundances of the proteolytic bacteria (*Bacillus megaterium* and *Staphylococcus cohnii*) were reduced significantly as compared to TC. Partial least-squares path modeling suggested that various physicochemical properties such as higher temperature as well as lower C/N ratio during composting played a dominant role in affecting the abundance of proteolytic bacteria, which may have been an important factor contributing to the lower NH_3_ loss in HPC. All these findings lead us to conclude that the HPC can significantly reduce NH_3_ loss by inhibiting the proteolytic bacteria and protease activity responsible for NH_3_ release.

**Graphical Abstract:**

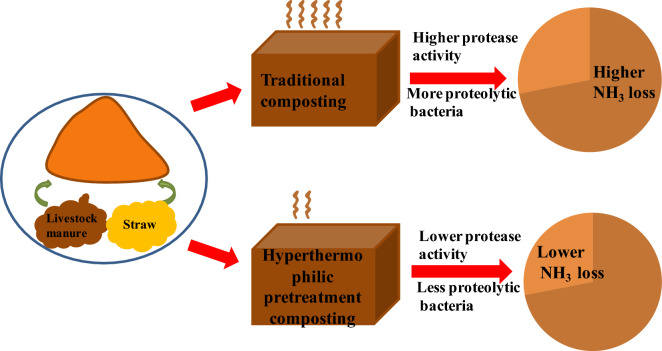

**Supplementary Information:**

The online version contains supplementary material available at 10.1186/s40643-023-00659-y.

## Keypoints

- Hyperthermophilic pretreatment composting (HPC) resulted in lower NH_3_ emissions .
- This is attributed to the decline in proteolytic bacteria and protease activity.
- Proteolytic bacteria was controlled by physicochemical properties in HPC.

**Supplementary Information:**

The online version contains supplementary material available at 10.1186/s40643-023-00659-y.

## Introduction

Composting is an important way to transform agricultural wastes into usable resources; however, it may result into the substantial nitrogen (N) loss through ammonia (NH_3_) volatilization (Shah et al. 2016). This can lead to generate secondary pollution and large amounts of nutrient loss from the organic material (Han et al. 2018). Thus, there is a dire need to explore the alternate composting methods to mitigate NH_3_ volatilization. This requires identifying the factors that control NH_3_ production from organic N mineralization and its emission.

During the composting of poultry manure, organic N mineralization occurs through a stepwise process including proteolysis, ammonification, and nitrification, which are primarily carried-out by microbial-derived extracellular enzymes (Ouyang and Norton 2020). Among these processes, proteolysis is considered the rate-limiting step in the ammonification process, as the protease is required to hydrolyze proteins into amino acids and subsequently metabolize resulting in NH_3_ volatilization and the release of NH_4_^+^ in the composts (Swelum et al. 2021). Previous studies have reported that the reduced NH_3_ loss is attributed to decreased protease activity during the composting (Cui et al. 2019; Xue et al. 2023). However, specific enzyme activities cannot distinguish the microorganisms immediately involved in degradation of diverse nitrogenous compounds in the composting process, so the relation between the microbial community and key enzymes remained poorly understood. Evaluation of functional genes encoding key enzyme may lead to revelation of the microbial communities involved in proteolysis and their relation to NH_3_ emission in composting systems (Nannipieri et al. 2012; Phillips et al. 2015).

Both bacteria and fungi have proteolytic activities, with Proteobacteria and Firmicutes containing members that play an important role in the N cycle (Hoang et al. 2022). For instance, *Pseudomonas* and *Bacillus* are widely distributed bacterial genus and have been found to express high proteolytic activity (Jurado et al. 2014). The bacterial species, *Pseudomonas fluorescens*, *Bacillus cereus* and *Bacillus mycoides* were cultivated and found to excrete metallopeptidases during culture (Bach and Munch 2000). It is well known that the predominant extracellular peptidases of bacterial origin are mainly alkaline metallopeptidases (Apr), neutral metallopeptidases (Npr) and serine peptidases (Sub) (Lori et al. 2020). The *apr*, *npr* and *sub* genes, which encode these extracellular peptidases that catalyze the proteolytic process, have been extensively used as marker genes to study the protease-encoding microbial communities (Pereg and McMillan 2020). For example, Sakurai et al. (2007) studied the diversity of *npr* after application of organic and inorganic fertilizer and they postulated that the proteolytic bacterial community composition may primarily contribute to control the overall soil protease activity. In the composting system, serine- and metalloproteases are the main proteolytic enzymes that are secreted by *Bacillus* isolates (Ramos et al. 2019). However, the knowledge in the dynamics of proteolytic bacteria is insufficient in the compost systems.

Compared to traditional composting (TC), the pretreatment of a hyperthermophilic reactor followed by TC (hyperthermophilic pretreatment composting, HPC) has been found to be superior in terms of compost quality and efficiency, as it accelerates humification of composts (Huang et al. 2019b), shortens the maturation period (Yamada et al. 2007, 2008) and increases the N retention (Huang et al. 2019a).Variations in the total bacterial community have been mainly linked to these advantages of HPC over TC (Huang et al. 2019a, 2019b). However, the differences in the proteolytic bacterial communities and their link to NH_3_ emissions between HPC and TC have not been further investigated.

In this study, we compared the effects of HPC and TC on (i) the amount of NH_3_ emissions; (ii) the dynamics of the proteolytic bacterial community participating in proteolysis in composting systems on the basis of the functional genes (*npr* and *sub*); (iii) the correlation between the proteolytic bacterial community, biophysiochemical characteristics, and NH_3_ emissions.

## Materials and methods

### Composting procedure and sampling

The HPC and TC composting piles were established according to the procedure outlined in Huang et al. (2019a). Briefly, each composting pile consisted of a mixture of 200 kg of pig manure and rice straw, with an initial C/N ratio of 25 and moisture content of 60%. For HPC, the mixture was stirred in a 400 L hyperthermophilic pre-treatment reactor and the temperature was artificially controlled via an oil bath heated up to 90 °C which sustained for 4 h. Then, the mixture was then transferred to the TC reactor after the temperature decreased to the ambient temperature. The TC reactor had a volume of 374 L with a height of 92 cm and an inner diameter of 72 cm. Triplicate samples from each reactor were collected on day 0, 20, 40 and 60 for biophysicochemical analyses and DNA extraction.

From both the composting systems, NH_3_ emissions were measured daily during the first two weeks and once every two days for the remaining composting period. At each NH_3_ measuring event, an air blower was used to remove some accumulated gas from the headspace and then a 10 cm plastic tube with a 2.5 mm diameter was fixed to the lid with screws and a rubber stopper to sample the gas from the headspace. Gas of 50 ml was sampled at 0, 30 and 60 min after closure.

### Analyses of compost

The composting temperature, pH, moisture, electrical conductivity (EC), total nitrogen (TN), total organic carbon (TOC), inorganic N (NH_4_^+^-N, NO_3_^−^-N), protease and ammonification rate were measuring following the procedures stated previously in Huang et al. (2019a). The N_2_O was determined using an Agilent 7890A gas chromatograph (Agilent, Beijing, China) (Huang et al. 2021). The concentration of NH_3_ was determined by absorbing NH_3_ with 2% boric acid and applying the titrimetric method as described by Cui et al. (2019). The detailed procedure for determination of water soluble organic N (WSON), the water-soluble N (WSN) and the calculation of the WSON are described in Xing et al. (2019).

### Analysis of proteolytic bacterial communities

DNA was extracted from soil using the FastDNA SPIN Kit (MP Biomedicals, Solon, OH, USA) according to the manufacturer’s instructions. For Illumina amplicon sequencing, primers targeting the proteolytic bacteria peptidase genes (*npr* (npr I/npr II) and *sub* (sub I/sub II) genes) (Bach et al. 2001) were modified to include Illumina adapter consensus at the 5’-ends of the forward and reverse primers to generate amplicons. The detailed PCR reactions, purification of amplicons, multiplexing, library pooling and sequencing process were previously described (Huang et al. 2018) and are shown in the Additional file 1. The sequencing results were deposited in the NCBI database with the accession numbers SRR13638639-SRR13638685, SRR13674937, SRR13675020 for proteolytic bacterial *npr* and *sub* genes.

### Statistical analyses

One-way analysis of variance (ANOVA) followed by Newman-Keuls multiple comparison test was conducted using SPSS 20.0 (SPSS Inc., Chicago, III,U.S.A.) and Origin Pro8G (OriginLab corporation, Northampton, USA) to evaluate significant differences (*p* < 0.05) between samples for biophysicochemical properties. Additionally, redundancy analysis (RDA) was performed using the Canoco5 program (Ter Braak and Smilauer 2002) to explore the relationships between proteolytic bacterial community structure and environment parameters. Lastly, partial least squares path modeling (PLS-PM) was used to evaluate the complex relationships among diverse variables on NH_3_ emissions using the R package plspm (v 0.4.7). The variables included in the model were biophysicochemical properties (pH, EC, DOC, TOC, TN, NH_4_^+^-N, NO_3_^−^-N, WSON, WSIN, protease activity), composting temperature, abundance of dominant proteolytic bacteria (relative abundances of *Bacillus megaterium*, *Staphylococcus cohnii*, *Bacillus subtilis* and *Novibacillus thermophilus*) and proteolytic bacterial community composition (based on OTU abundances).

## Results

### Variations of NH_3_ emissions between different treatments

**Fig. 1:**
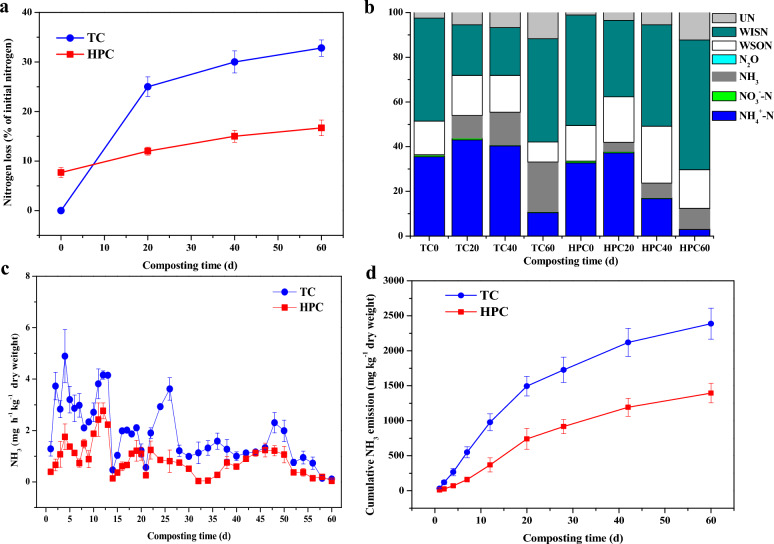
The nitrogen loss (**a**), nitrogen balance (**b**), NH_3_ flux rate (**c**) and cumulative NH_3_ emission (**d**) during traditional composting (TC) and hyperthermophilic pretreatment composting (HPC). The “UN”represent unclassified nitrogen, the WISN represent the water-insoluble nitrogen, the WSON represent the water soluble organic nitrogen

Results revealed that 33% of the initial TN was lost during 60 days from TC (Fig. 1a), whereas the N loss fraction in case of HPC was only 17%, indicating 49% lower N loss from HPC as compared to TC. The NH_3_ emissions was peaked during the thermophilic phases (> 55 °C, Additional file 1: Fig. S1) for TC (Fig. 1c), ranging from 0.46 to 4.89 mg kg^−1^ dry weight. At the same stage, the NH_3_ flux rate for HPC ranged from 0.14 to 2.76 mg kg^−1^ dry weight which was significantly (*p* < 0.05) lower than TC. The cumulative NH_3_ emission in HPC was 42% lower as compared to TC (1.39 vs. 2.38 g kg^−1^ dry weight) (Fig. 1d). The NH_3_ emission was accounted for 22% of the TN loss over 60 days from HPC whereas the respective fraction in case of TC was 45% (Additional file 1: Table S2). However, there existed a minor difference in the cumulative N_2_O emissions as percent of TN loss for both the treatments (< 0.1%, Fig. 1b).

### The proteolytic bacterial diversity between different treatments

**Fig. 2:**
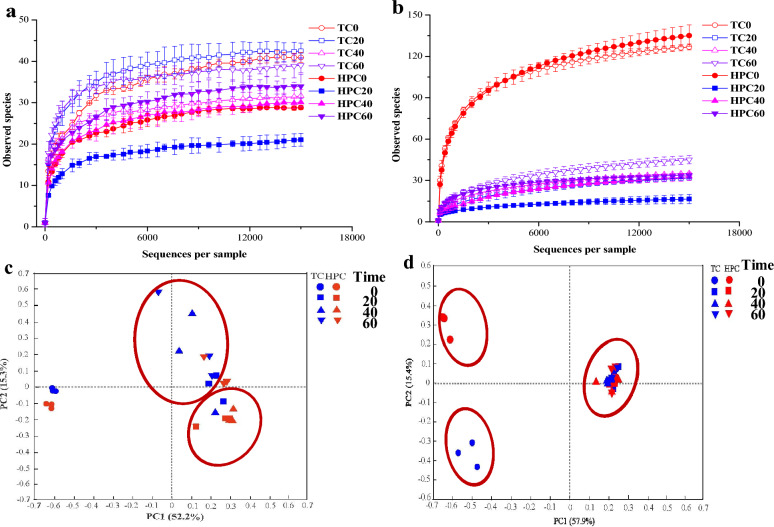
Rarefraction curve of observed *npr* (**a**) and *sub* (**b**) OTUs and the overall distribution of OTU-based *npr* (**c**) and *sub* (**d**) community dissimilarity during traditional composting (TC) and hyperthermophilic pretreatment composting (HPC) (based on unweighted UniFrac PCoA results). The number 0, 20, 40 and 60 represent different composting time. Different colors indicate composting days

For each sample, an average of 23,013 and 21,165 quality reads were obtained for *npr* and *sub* genes, respectively (Additional file 1: Table S3). Notably, the number of *sub* gene OTUs (32 to 144 OTUs g^−1^ dry soil) was several times higher than that of *npr* gene OTUs (20 to 42 OTUs g^−1^ dry soil). In terms of the microbial community structure, distinct *npr* communities were seen among the two treatments during composting (Fig. 2c) whereas the *sub* community showed a similar community distribution in the later period (day 20–60) of the composting (Fig. 2d). Additionally, HPC was found to have a lower microbial richness (Chao 1) and diversity (Shannon index) for both *npr* and *sub* communities compared to TC (Additional file 1: Table S3).

**Fig. 3:**
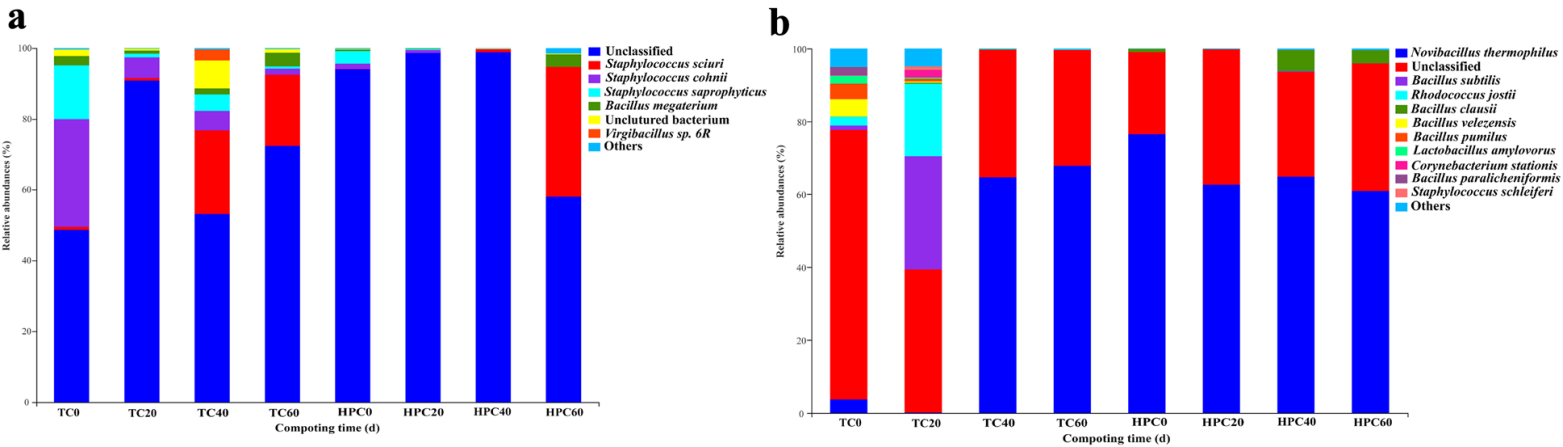
Taxonomic classification at the species level of abundant (> 1%) *npr* and *sub* gene sequences in the samples during the traditional composting (TC) and hyperthermophilic pretreatment composting (HPC) by day 0, 20, 40 and 60. The prefix “Un.” represented unclassified species

The majority of the *npr* and *sub* gene sequences belonged to Firmicutes in both HPC (0.98–49.5%) and TC (19.7–77.4%) (data not shown). The relative abundances of the proteolytic bacteria community at the genus- and species-level were compared in Additional file 1: Fig. S3 and Fig. 3, respectively. During the period of day 0–40, the most dominant *npr* gene OTUs for TC belonged to *Staphylococcus* (7.6–46.8%) and were found to be 1.4 to 50 times higher compared to HPC. For *both* genes, *Bacillus* was more abundant in TC than that in HPC (Additional file 1: Fig. S3). Following this, the relative abundance of *Bacillus megaterium* and *Staphylococcus cohnii*, accounted for 6% to 33% of all the *npr* gene sequences in TC, which was higher than HPC (Fig. 3a). In addition, the relative abundance of *Novibacillus* was seen to be prevalent throughout composting period in HPC, while it was observed to dominate by day 40–60 in TC.

### Correlations between environmental parameters and proteolytic bacteria

**Fig. 4:**
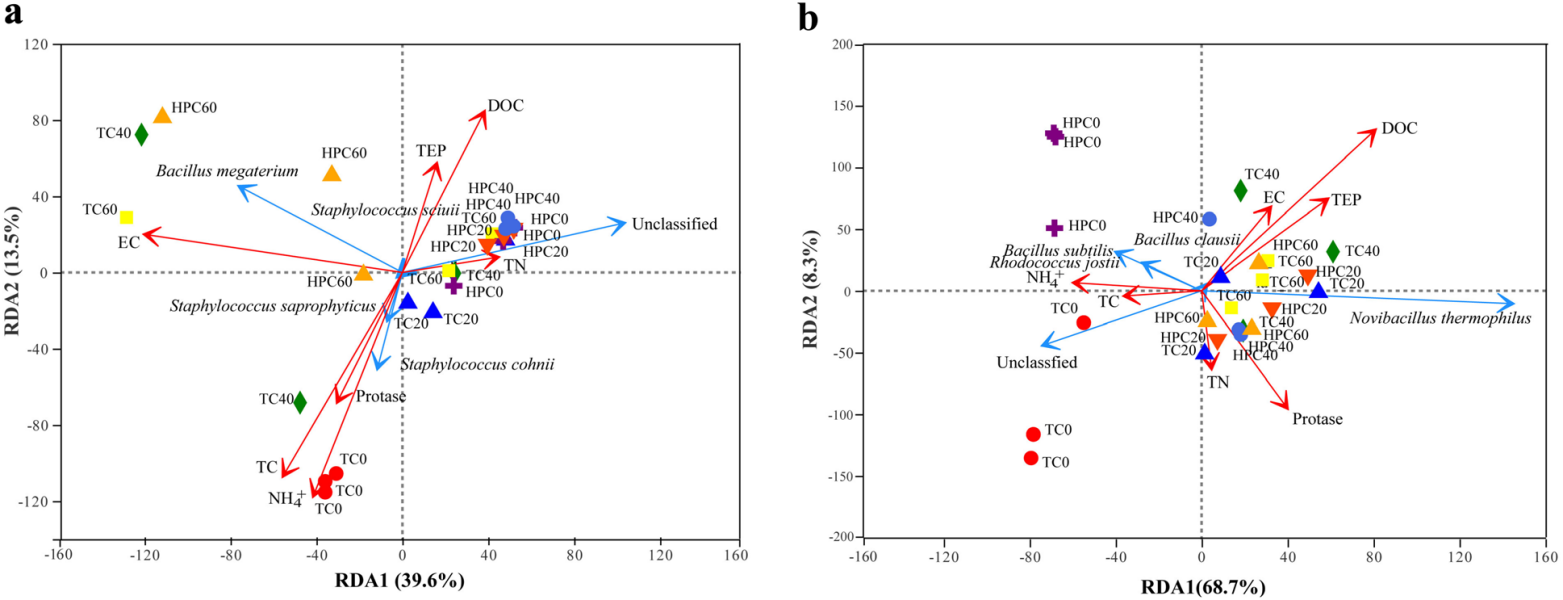
Redundancy analysis (RDA) between proteolytic bacterial *npr* (**a**) and *sub* (**b**) taxa (relative abundances of the dominant species at top 5) and environmental parameters (TOC, TN, EC, DOC, TEP, NH_4_^+^ and Protase) in the TC (traditional composting) and HPC (hyperthermophilic pretreatment composting) by Day 0, 20, 40 and 60. TOC: total organic carbon, TN: total nitrogen, EC: electricity conductivity, DOC: dissolved organic carbon, TEP: temperature. The prefix “Unclassified” represented unclassified species

In this study, RDA analysis was conducted to investigate the influence of 7 physicochemical properties (TOC, TN, EC, DOC, TEP, NH_4_^+^ and protease activity) on the *npr* and *sub* community structures in HPC and TC. The Adonis analysis indicated that these structures were significantly (*p* < 0.05) affected by the composting treatments. Additionally, the first two axes of the RDA explained 40% and 69% of the variation in the abundances of *npr* (Fig. 4a) and *sub* (Fig. 4b) taxa, respectively. Furthermore, DOC, total C and NH_4_^+^-N were identified as the most significant (*p* < 0.01) environmental factors affecting the proteolytic bacterial community compositions, followed by temperature. When taking a closer look at the individual taxa, NH_4_^+^-N, protase activity and TOC were significantly positively related to *Staphylococcus cohnii*, while temperature was negatively correlated with *Bacillus megaterium*. As for *the sub* taxa, *Novibacillus thermophilus* was found to accumulate in HPC and exhibited a positive correlation to TN.

### The selected factors contributing to NH_3_ emissions between different treatments

**Fig. 5:**
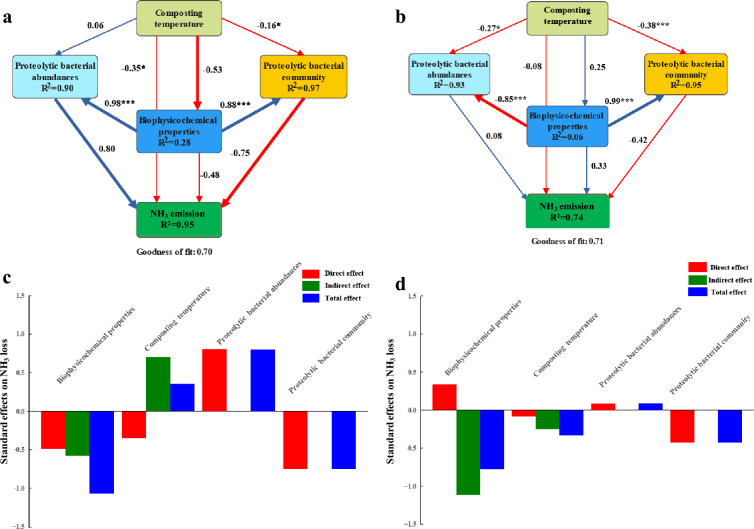
Direct and indirect effects of different factors on TN loss for traditional composting (TC) and hyperthermophilic pretreatment composting (HPC). PLS-PM showing the relationships among biophysicochemical properties, proteolytic bacterial abundance and proteolytic bacterial community with respect to TN loss for TC (**a**) and HPC (**b**). The positive and negative effects are indicated by blue and orange arrows, respectively. Larger path coefficients are indicated by wider arrows, red and blue colors indicate positive and negative effects, respectively. Significance levels of Path coefficients are indicated by ^*^ (*p* < 0.05) and ^***^(*p* < 0.001). Standardized direct and indirect effects estimated by PLS-PM for TC (**c**) and HPC (**d**)

A PLS-PM was used to reveal the relationship among biophysicochemical properties, composting temperature, proteolytic bacterial abundances and community with respect to the NH_3_ emissions for HPC and TC, respectively. As indicated in Fig. 5a, proteolytic bacterial abundance had an accelerating effect on NH_3_ emissions in HPC and TC but to a lesser degree in HPC. Furthermore, biophysicochemical properties had a negative total effect on NH_3_ emissions for TC and HPC (Fig. 5c and d), even though with different magnitude. Correspondently, the abundances of proteolytic bacteria were strongly inhibited by biophysicochemical properties including composting temperature in HPC as compared to TC (Fig. 5a and b).

## Discussions

### Hyperthermophilic pretreatment composting decreases TN loss

The HPC resulted in a 42% reduction in NH_3_ emission than the TC, which in turn substantially minimized the TN loss during the composting period. This was in consistent with some previous studies (Pan et al. 2018; Cao et al. 2018). Although high temperature during composting is usually considered to increase NH_3_ volatilization (Koyama et al. 2018), the higher temperature during the thermophilic phase for HPC reduced the NH_3_ emissions. This may be attributed to the decrease in protease activity for HPC (Additional file 1: Fig. S2a). Protease plays an important role in the hydrolysis catalyzation to produce NH_3_ (Neemisha and Sharma 2022). Previous studies have shown that the proteolytic activity in the leachate of compost piles is thermally stable up to 60 °C, but it is reduced by 50% when temperature exceed 65 °C (Ramos et al. 2019). In our study, the temperature in the thermophilic stage for TC remained below 60 °C, while the temperature in the same stage of HPC exceeded 65 °C and was sustained for a longer period of time (10 days). This difference of temperature profiles may largely explain the lower proteolytic activity and the reduced NH_3_ volatilization in HPC.

Additionally, on day 60, WSON content in HPC comprised 17% of the TN content, while this fraction was only 9% for TC (Fig. 1b). The higher content of WSON in HPC may have contributed to the reduced ammonification activity (Additional file 1: Fig. S2b), in evidenced by a previous study (Cui et al. 2019). Variations in physicochemical properties, including composting temperature, pH, EC, TN, NH_4_^+^-N, and NO_3_^−^-N are detailed in the previous study (Huang et al. 2019a) and can be found in the supporting information.

### Hyperthermophilic pretreatment composting reduces proteolytic bacterial diversity

The lower microbial richness and diversity observed for both *npr* and *sub* communities in HPC was consistent with changes in bacterial communities targeting the bacterial 16S rRNA gene (Huang et al. 2019a). Thus, the protease activity may be negatively affected by decreased proteolytic bacterial communities. For TC, the most dominant *npr* gene OTUs belonged to *Staphylococcus*, which has been identified as ammonification bacteria in a previous study (Xiao et al. 1995). *Bacillus megaterium* was reported to be a widely distributed ammonifier with high proteolytic capability (Jurado et al. 2014). Correspondently, the relative abundance of *Bacillus megaterium* and *Staphylococcus cohnii* for TC were higher compared to HPC. Thus, the decrease in relative abundance of the proteolytic bacteria was likely beneficial for NH_3_ mitigation (Cui et al. 2019) in HPC. Additionally, the relative abundance of *Novibacillus thermophilus*, which is known to participate in the biodegradation of organic matter (Wang et al. 2020) and is moderately thermophilic within the family *Thermoactinomycetaceae* (Yang et al. 2015), was significantly higher throughout HPC. This microorganism has been reported to increase the TN content of composts (Li et al. 2020; Wan et al. 2021), which may also contribute to lower N loss in HPC. However, further research is needed to investigate the exact mechanism behind the contribution of *Novibacillus thermophilus* to the lower N loss in HPC.

### Correlations between environmental parameters and proteolytic bacteria

A previous study has shown that *Bacillus megaterium* produces proteases and has a higher proteolytic activity compared to other microbes during the composting period (Jurado et al. 2014). In this study, a negative correlation between temperature and *Bacillus megaterium* was observed for *npr* taxa. Therefore, the high temperature in HPC may have a detrimental effect on the abundance and activity of the proteolytic bacteria, which may explains the lower N loss compared to TC (Huang et al. 2019a). For *sub* taxa, an accumulation of *Novibacillus thermophilus* was observed in HPC and it is found to have a positive correlation with TN, consistent with previous studies in composting systems (Li et al. 2020; Wan et al. 2021).

### Relative contributions of selected factors to NH_3_ emissions

The results of PLS-PM analysis indicated that proteolytic bacterial abundance had a significant effect on NH_3_ emissions in HPC and TC but to a lesser degree in HPC. This supports the findings of previous studies which have suggested that microbial abundance is a crucial factor in controlling NH_3_ emissions throughout composting system (Cui et al. 2019). Additionally, biophysicochemical properties have been reported to be primary factors affecting NH_3_ emissions during composting (Cao et al. 2020). In this particular study, the total effect of biophysicochemical properties on N loss was found to be negative for both treatments. This could be attributed to the fact that the biophysicochemical can influence the microbial abundances and community structure (Cao et al. 2020). For instance, the abundances of proteolytic bacteria were seen to be significantly inhibited by biophysicochemical properties in HPC such as the higher composting temperature and lower C/N ratio compared to TC. This result is further supported by our previous study which suggested that biophysicochemical properties in HPC could heavily suppress the growth and activity of ammonifiers (Huang et al. 2019a), and thereby resulting in lower NH_3_ emissions.

## Conclusion

In this study, HPC was able to reduce N loss by 49% through inhibition of NH_3_ emissions (42%) during 60 days of composting period compared to TC. The lower NH_3_ emissions were likely due to the decrease in protease activity, ammonification rate and proteolytic bacterial population in HPC. Additionally, biophysicochemical properties such as higher temperature and lower C/N ratio could have greatly suppressed the growth and activity of proteolytic bacteria, resulting in lower NH_3_ emissions. This study has provided further insights into the application potential of HPC for NH_3_ mitigation and the role of proteolytic bacteria in controlling the NH_3_ emissions.

### Supplementary Information

**Additional file 1: Table S1.** Biophysicochemical properties. **Table S2.** Total N loss as NH_3_ from different treatments during composting. **Table S3.** Illumina amplicon sequencing reads and comparison of α-diversity indices of the bacterial *npr* and *sub* communities in the traditional composting (TC) and hyperthermophilic pretreatment composting (HPC). Different letters indicated statistical significance (*p *< 0.05) based on Newman-Keuls multiple comparison test. **Figure S1**. Temperature profiles of two composting treatments over 60 days. TC, traditional composting; HPC, hyperthermophilic pretreatment composting. **Figure S2.** The protease activity (a) and ammonification rate (b) of two composting treatments over 60 days. TC, traditional composting; HPC, hyperthermophilic pretreatment composting. **Figure S3.** Taxonomic classification at the genus level of abundant (> 1%) npr and sub gene sequences in the samples during the traditional composting (a) and hyperthermophilic pretreatment composting (b) by day 0, 20, 40 and 60. The prefix“Un.”represented unclassified species.

## Data Availability

All data generated or analyzed during this study are included in this published article (and its Additional file 1).
