# Supplementary material for: Hyperthermophilic pretreatment composting can reduce ammonia emissions by controlling proteolytic bacterial community and the physicochemical properties
Source: Bioresour Bioprocess. 2023 Jul 8;10(1):37. doi: 10.1186/s40643-023-00659-y (PMC10992325; doi:10.1186/s40643-023-00659-y)
Supplement: Supplementary file 1 — Additional file 1: Table S1. Biophysicochemical properties. Table S2. Total N loss as NH3 from different treatments during composting. Table S3. Illumina amplicon sequencing reads and comparison of α-diversity indices of the bacterial npr and sub communities in the traditional composting (TC) and hyperthermophilic pretreatment composting (HPC). Different letters indicated statistical significance (p < 0.05) based on Newman-Keuls multiple comparison test. Figure S1. Temperature profiles of two composting treatments over 60 days. TC, traditional composting; HPC, hyperthermophilic pretreatment composting. Figure S2. The protease activity (a) and ammonification rate (b) of two composting treatments over 60 days. TC, traditional composting; HPC, hyperthermophilic pretreatment composting. Figure S3. Taxonomic classification at the genus level of abundant (> 1%) npr and sub gene sequences in the samples during the traditional composting (a) and hyperthermophilic pretreatment composting (b) by day 0, 20, 40 and 60. The prefix“Un.”represented unclassified species. [file 40643_2023_659_MOESM1_ESM.docx]

**Additional file**

**Hyperthermophilic pretreatment composting can reduce ammonia emissions by controlling proteolytic bacterial community and the physicochemical properties**

Ying Huang^a .^ Yuehong Chen^a*.^ Hongying Huang^bc.^ Ghulam Mustafa Shah^d .^Jiujun Lin^a^ Meiling Yan^a .^Chengbao Guo^a .^ Xu Xiao ^a^

(^a^*Nanjing Institute of Agricultural Sciences in Jiangsu Hilly Area, No. 6 Xianyin South Road, Qixia District, Nanjing 210046, Jiangsu Province, China;*

^b^*Institute of Agricultural Resources and Environment Jiangsu Academy  of Agricultural  Sciences, No. 50 Zhongling Street, Xuanwu District, Nanjing, Jiangsu Province 210014, China;*

^c^*Jiangsu Collaborative Innovation Center for Solid Organic Waste Resource Utilization, Nanjing, Jiangsu Province 210014, China；*

^d^*Department of Environmental Sciences, COMSATS University Islamabad Vehari Campus, Vehari 61100, Pakistan*)

*Correspondence should be addressed to

Yuehong Chen

e-mail address: cyh2007104026@163.com

**Main content**

Table S1 to Table S3

Figure S1 to Figure S3

Table S1 Biophysicochemical properties

|  |  | Physicochemical properties of composts | | | | | | | | |
| --- | --- | --- | --- | --- | --- | --- | --- | --- | --- | --- |
| Treatment | Time | TOC | TN | pH | EC | NH_4_^+^-N | NO_3_^-^-N | WISN | WSON | DOC |
|  | (d) | (g/kg) | (g/kg) |  | (ms/cm) | (mg/kg) | (mg/kg) | (g/kg) | (g/kg) | (mg/kg) |
| CK | 0 | 379.9±10.8**a** | 20.4±2.3**bc** | 8.9±0.1a**b** | 34.1±0.6 **bc** | 7160±272 **a** | 159.1±13.6 **a** | 9.2±3.4**a** | 3.0±3.7**c** | 149.1±47.2**bc** |
|  | 20 | 378.8±1.8**a** | 18.1±0.2**d** | 8.0±162**c** | 46.8±3.6 **a** | 7278±210**a** | 115.8±11.4 **b** | 5.3±1.1**c** | 4.4±2.5**e** | 183.9±18.9**ab** |
|  | 40 | 354.8±30**bc** | 16.3±0.6**d** | 8.8±119 **b** | 36.1±0.5 **b** | 5951±213 **b** | 61.8±10.5 **e** | 4.1±1.4**c** | 3.1±0.4**d** | 150.7±62.7**b** |
|  | 60 | 275.9±5.3**d** | 17.6±0.4**d** | 9.3±194**a** | 32.3±0.8**bc** | 1836±477 **d** | 87.6±2.3 **d** | 10.0±1.5**b** | 2.0±0.5**b** | 102.7±30.8**c** |
| HPC | 0 | 380.3±6.3 a | 18.4±0.1cd | 8.8±0.1b | 31.6±0.9c | 6019±273b | 147.8±14.0a | 9.8±1.9b | 3.4±1.4bc | 154.9±10.2b |
|  | 20 | 367.6±19.7ab | 21.1±2.4b | 8.7±0.2b | 34.4±1.5b | 6435±111b | 107.1±13.8bc | 8.2±0.2c | 4.7±2.3e | 171.5±33.8a |
|  | 40 | 339.0±5.0 c | 25.1±1.2 a | 9.2±0.1a | 35.2±1.0b | 2905±186c | 84.1±11.7d | 11.9±1.6b | 5.5±3.6bc | 123.8±27.4a |
|  | 60 | 288.6±9.3 d | 24.4±0.5a | 9.4±0.1a | 31.9±1.3c | 710±163e | 92.3±6.9cd | 14.7±1.1a | 4.1±3.9a | 95.9±28.6c |

Table S2 Total N loss as NH_3_ from different treatments during composting

| Treatments | Initial TN (kg) | Total N after pretreatment (kg) | Total N lost after pretreatment (kg) | Total N at end  (kg) | Total N lost in all (kg) | Total N lost (%) | Total N retained in compost (% of initial N) | NH_3_ emission (kg) | Total N lost as NH_3_ (% of TN lost) |
| --- | --- | --- | --- | --- | --- | --- | --- | --- | --- |
| TC | 1.68±0.05 | 1.68±0.05 | / | 1.13±0.02 | 0.55±0.01 | 32.8±2.5 | 67.2±3.2 | 0.25±0.02 | 45.4±2.1 |
| HPC | 1.68±0.05 | 1.55±0.07 | 0.13±0.01 | 1.40±0.03 | 0.28±0.02 | 16.7±1.1 | 83.3±5.1 | 0.12±0.01 | 21.8±1.5 |

Abbreviations: TC, traditional composting; HPC, hyperthermophilic pretreatment composting.

Table S3 Illumina amplicon sequencing reads and comparison of α-diversity indices of the bacterial *npr* and *sub* communities in the traditional composting (TC) and hyperthermophilic pretreatment composting (HPC). Different letters indicated statistical significance (*p* < 0.05) based on Newman-Keuls multiple comparison test.

|  | Treatment | Number of reads | Richness estimator | | Diversity estimator | | |
| --- | --- | --- | --- | --- | --- | --- | --- |
|  |  | Filtered data | Observed OTUs | Chao1 | Shannon | Simpson |  |
| *npr* | TC0 | 23705±385 | 36±3.2**bc** | 42±1.3**a** | 1.55±0.02a**b** | 0.29±0.002**e** |  |
|  | TC20 | 21409±1571 | 30±3.0**cd** | 37±1.7**b** | 1.59±0.09**ab** | 0.43±0.018**c** |  |
|  | TC40 | 23548±390 | 40±3.6**ab** | 36±2.1**b** | 1.36±0.11**b** | 0.35±0.012**de** |  |
|  | TC60 | 19832±2466 | 33±2.9**bcd** | 37±1.8**b** | 1.66±0.12**a** | 0.31±0.029**e** |  |
|  | HPC0 | 23748±1030 | 31±2.1cd | 28±0.6d | 0.58±0.19c | 0.77±0.062b |  |
|  | HPC20 | 23408±1002 | 20±1.8e | 22±0.7e | 0.45±0.07c | 0.80±0.023a |  |
|  | HPC40 | 24029±480 | 28±2.3d | 32±2.7c | 0.56±0.12c | 0.78±0.071ab |  |
|  | HPC60 | 24432±526 | 42±3.7a | 44±3.0a | 1.39±0.03b | 0.39±0.033cd |  |
|  | *Total sequences* | *184114* |  |  |  |  |  |
| *sub* | TC0 | 22924±1020 | 129±4.5**b** | 132±6.2**b** | 3.06±0.18**a** | 0.10±0.01**b** |  |
|  | TC20 | 21336±3712 | 34±2.0**d** | 34±0.17**de** | 1.09±0.11**c** | 0.47±0.02**a** |  |
|  | TC40 | 18723±1054 | 32±0.7**d** | 35±3.1**de** | 0.81±0.07**d** | 0.65±0.05**a** |  |
|  | TC60 | 19920±1959 | 49±4.2**c** | 56±4.2**c** | 1.18±0.02**c** | 0.46±0.05**a** |  |
|  | HPC0 | 23519±1959 | 144±3.5a | 145±12.0a | 2.78±0.01b | 0.15±0.05b |  |
|  | HPC20 | 22455±500 | 25±2.1e | 26±1.1e | 1.04±0.12cd | 0.50±0.03a |  |
|  | HPC40 | 19060±4642 | 33±1.4d | 40±2.5de | 1.12±0.04c | 0.46±0.03a |  |
|  | HPC60 | 21385±7557 | 36±1.0d | 44±1.4cd | 1.11±0.09c | 0.47±0.01a |  |
|  | *Total sequences* | *169325* |  |  |  |  |  |

**Fig. S1** Temperature profiles of two composting treatments over 60 days. TC, traditional composting; HPC, hyperthermophilic pretreatment composting.

**Fig. S2** The protease activity (a) and ammonification rate (b) of two composting treatments over 60 days. TC, traditional composting; HPC, hyperthermophilic pretreatment composting.

**Fig. S3** Taxonomic classification at the genus level of abundant (> 1%) *npr* and *sub* gene sequences in the samples during the traditional composting (a) and hyperthermophilic pretreatment composting (b) by day 0, 20, 40 and 60. The prefix“Un.”represented unclassified species.

Figure S1 Temperature profiles of two composting treatments over 60 days.

TC, traditional composting; HPC, hyperthermophilic pretreatment composting.

Figure S2 The protease activity (a) and ammonification rate (b) of two composting treatments over 60 days.

TC, traditional composting; HPC, hyperthermophilic pretreatment composting.

Figure S3 Taxonomic classification at the genus level of abundant (> 1%) *npr* and *sub* gene sequences in the samples during the traditional composting (a) and hyperthermophilic pretreatment composting (b) by day 0, 20, 40 and 60. The prefix“Un.”represented unclassified species.
